# Catalyzed and uncatalyzed procedures for the syntheses of isomeric covalent multi-indolyl hetero non-metallides: an account

**DOI:** 10.3762/bjoc.17.137

**Published:** 2021-08-19

**Authors:** Ranadeep Talukdar

**Affiliations:** 1 Department of Chemistry, Indian Institute of Technology Kharagpur, West Midnapore, West Bengal – 721302, India

**Keywords:** bisindole, heteroatom, indole, selenide, sulfide

## Abstract

Two or more indole molecules tailored to a single non-metal central atom, through any of their C2–7 positions are not only structurally engaging but also constitute a class of important pharmacophores. Although the body of such multi-indolyl non-metallide molecules are largely shared to the anticancer agent bis(indolyl)methane, other heteroatomic analogs also possess similar medicinal properties. This concise review will discuss various catalytic and uncatalytic synthetic strategies adopted for the synthesis of the non-ionic (non-metallic) versions of these important molecules till date.

## Introduction

Indole can be considered as a “prodigy” in the family of nitrogen-based heterocycles, because of its diverse presence in bioactive molecules [1–8], coupled with the distinct nucleophilic chemistry revolving its aromatic benzo-fused pyrrole system as encountered throughout the bibliography [9–15]. It is therefore obvious that a non-metal hydride will become exceptionally crucial when its hydrogen atoms are replaced by this special heterocycle, forming a multi-indolyl hetero non-metallide. In contemporary period, the said molecules have earned extensive importance in pharmacology to prevent cancer of a number of human organs, certified by the recent flooding of scientific literature related to bis(indolyl)methanes, which shows the usefulness of this class of molecules for prevention of this terminal disease [16–23]. Related molecules consisting of heteroatoms at the central tethering position have also appeared in the spotlight of anticancer research recently. In line with this high importance associated with the molecules of current topic, i.e., more than one indole molecule flanked by a central atom, conglomeration of the available synthetic methods will have a high scientific value. This review will give a concise account of the same, although preparations of ionic bis(indolyl) metal salts will not be considered [24–33].

## Review

### The pyrrole C2 and C3 linkages

By virtue of the two available sites in its pyrrole substructure, two indoles can be attached to a central atom via their C-2 or C-3 positions in a symmetric way. The non-symmetric variety may connect them with C-2 of one with the C-3 of another, via the central atom. Below described are such synthetic strategies which are classified depending on the central tethering atom, largely with boron, carbon, nitrogen, oxygen, silicon, phosphorus, sulfur, selenium, and tellurium. This review will skip the reports on the corresponding carbon-centered analogs.

#### Boranes

**Scheme 1:**
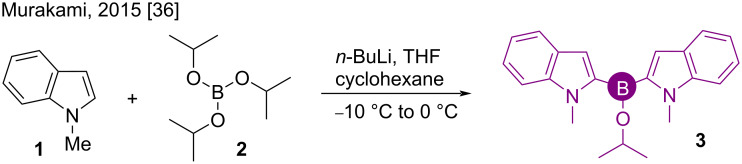
Synthesis of 2,2’-bis(indole)borinic ester **3**.

First discovered in 1894 [34], 2,2’-bisarylborinates are used for treating prostate cancers utilizing their property of inhibiting the transient receptor potential channels such as TRPM-7 [35]. In 2015, Murakami synthesized the novel indole C-2 borinic acid derivative **3** by reacting *N*-methylindole (**1**) with triisopropyl borate (**2**) in a strongly basic medium (Scheme 1). The product formation proceeds through the indole C-2 deprotonation mechanism [36].

**Scheme 2:**
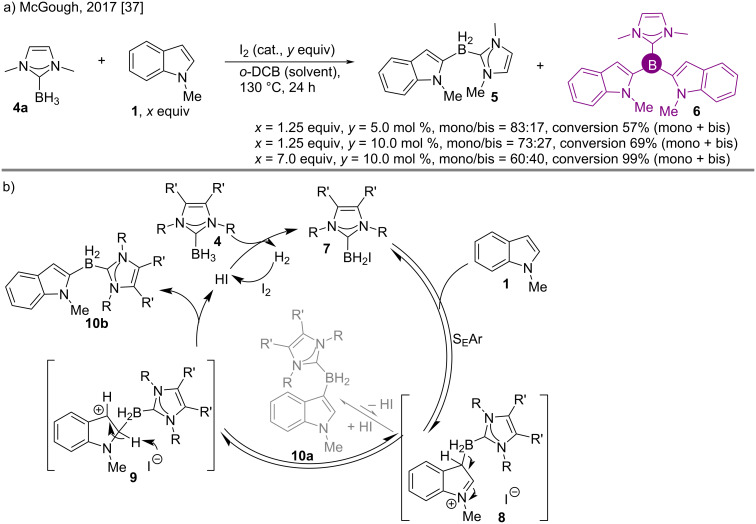
Synthesis of 2,2’-bisindole NHC**·**boranes by an S_E_Ar mechanism.

The reason behind the C-2 attachment of the boron atom rather than at the C-3 position of the indole ring was explained by McGough et al. [37]. They performed a base-free catalytic I_2_-assisted indole C–H functionalization (electrophilic borylation) using the *N*-protected indole **1** and NHC·borane **4a** that gave a mixture of the mono and bis isomers (**5** and **6**, respectively) in fair to excellent yields (Scheme 2a). Increasing the amount of iodine led to less unreacted starting material **1**, and increased formation of the bisindole product **6**. An almost quantitative conversion of **1** was observed with a high excess of the indole reactant.

It is seen that in the presence of a base the C-2 deprotonation becomes very fast in **9** (for regaining aromaticity) so the boron at the initial C-3-borylated intermediate **8** (formed via S_E_Ar) cannot migrate fast enough, leading to a C-3 borylation product **10a** (unlike Pd) [38–40]. Here the absence of the base resulted in a slow or no C-2 deprotonation of **9**, which in turn forces the boron to migrate to C-2 from C-3 (**8**, Scheme 2b) to result in the C-2 borylation (**10b**).

#### Amines

**Scheme 3:**
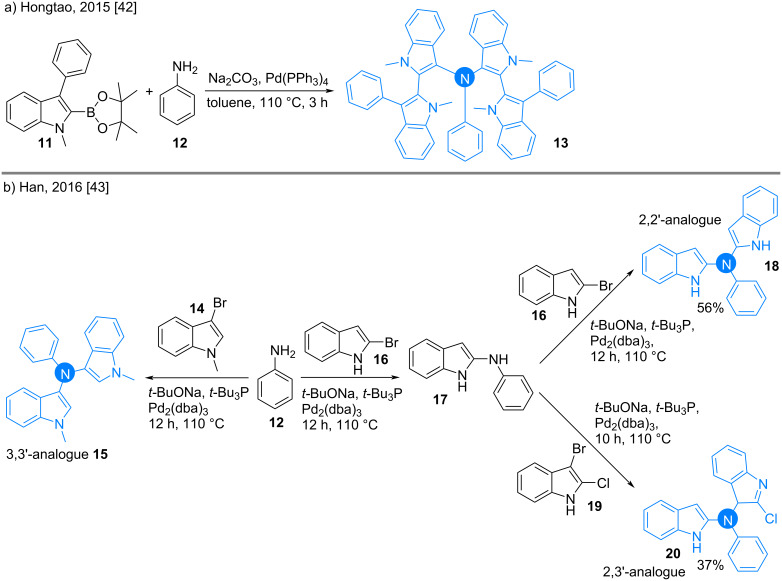
Syntheses of indolyl amines through Buchwald–Hartwig cross coupling.

Bis(indolyl)amines have recently become important as organic electroluminescent materials [41]. Hongtao and co-workers reported the synthesis of tetrakisindole species **13** through the coupling of aniline (**12**) and indole-2-boronic acid pinacol ester **11** using the Buchwald–Hartwig method (Scheme 3a) [42]. In a similar fashion, Han reported the syntheses of the symmetric and unsymmetric triaryl-substituted amines **15**, **18**, and **20** [43]. Taking aniline as the pivotal moiety, it was coupled with isomeric bromoindoles **14** and **16** for the synthesis of the targeted products (Scheme 3b).

#### Ethers

**Scheme 4:**
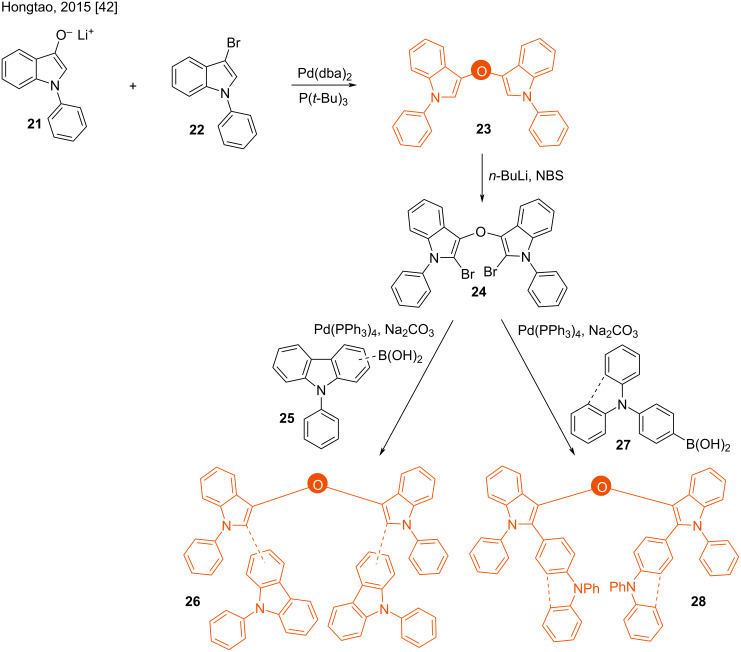
Synthesis of 3,3’-bis(indolyl) ethers.

Hongtao and co-workers also studied the electroluminescence properties of the 3,3’-bis(indolyl) ether derivatives **23**, **26**, and **28**. The materials were prepared by the Pd(0)-mediated coupling of lithium *N*-arylindole-3-alkoxide **21** with 3-bromo-*N*-arylindole **22**, followed by a further C-2 bromination (**24**) and subsequent Suzuki reaction with boronic acids **27** or **25** (Scheme 4) [42]. A similar class of molecules have found broad applications in organic electroluminescent devices [44].

#### Silanes

Heteroaryl compounds containing silicon, an earth abundant and non-toxic element, are important in organic electronics or photonics and in the field of drug discovery and nuclear medicine [45–50].

**Scheme 5:**
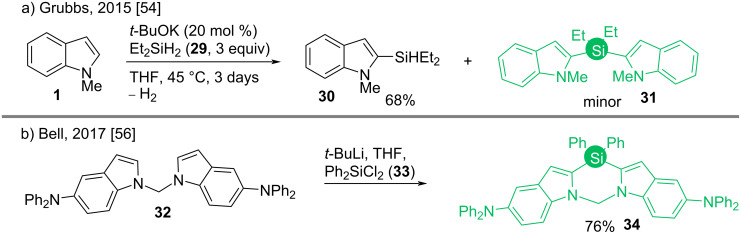
C–H silylation of indoles.

The first property could be attributed to the facile orbital interactions of the σ* orbital of silicon and the π* orbital of the butadiene unit, which overall lowers the energy of the LUMO [51–52]. Known previously with expensive transition-metal catalyst (Ru) [53], Grubbs demonstrated the first KO*t*-Bu-catalyzed C2–H silylation of *N-*methylindole (**1**) with observed H_2_ evolution [54]. Here the di(indol-2-yl)silane (**31**) was found as a minor product though (Scheme 5a). The reaction has a high turnover number of 92 and it was halted in the presence of radical scavengers. However, the mechanism was unidentified, although it was proved to not going via a Minisci-type silyl radical addition [55], as the reaction with pyridine did not afford any product.

Bell studied the properties of such molecules which are similar to those used in OLED devices (organic light emitting diodes) in 2017. The molecule **34** was synthesized by base-mediated reaction of bisindole derivative **32** with Ph_2_SiCl_2_ (**33**, Scheme 5b) [56]. The dissociation of the indole C-2–Si bond upon UV light excitation generates a hole transport layer (HTL) in these materials, facilitating the optical activity [57].

**Scheme 6:**
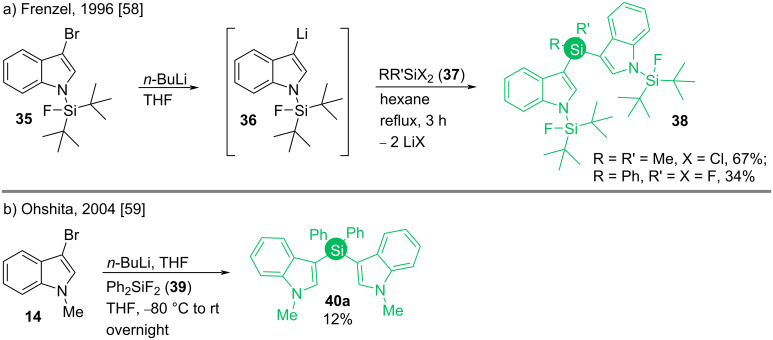
*n*-BuLi-mediated syntheses of bis(indol-3-yl)silanes.

In 1996, Frenzel reported the synthesis of bis(indol-3-yl)silane **38** that involved *n*-BuLi as the base [58]. The strategy was later adopted by Ohshita in 2004 (**40a**, Scheme 6) [59].

**Scheme 7:**
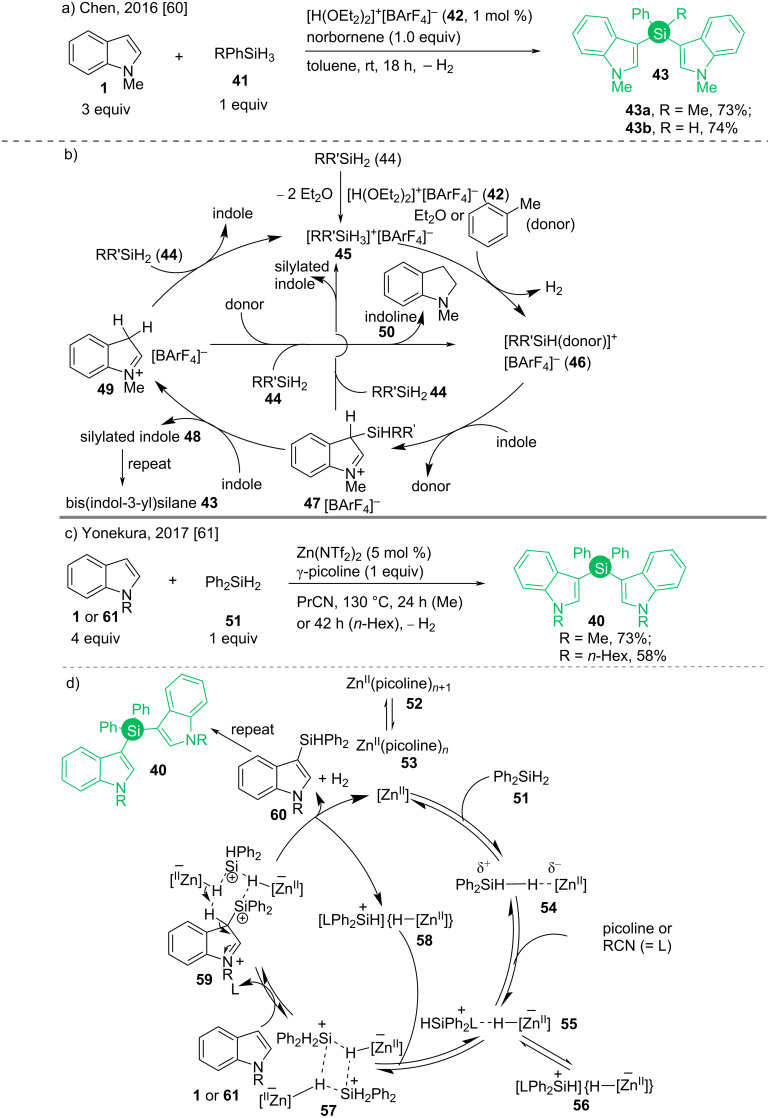
Acid-catalyzed syntheses of bis(indol-3-yl)silanes and mechanisms.

Between 2016 and 2018, some acid-catalyzed syntheses of bis(indol-3-yl)silanes appeared [60–63]. Chen and co-workers demonstrated a Brønsted acid-catalyzed Friedel–Crafts process, where hydrosilanes **41** were treated with an excess amount of indoles (Scheme 7a and Scheme 7b) [60]. Brookhart’s acid [H(OEt_2_)_2_]^+^[BAr^F^_4_]^−^ (**42**) was used to generate ether-stabilized silicon cations of type **46** and norbornene was added as a proton scavenger [64]. Following this procedure, Yonekura synthesized the similar compound **40**, using a catalytic Lewis acid Zn(NTf_2_)_2_ and stoichiometric Lewis base γ-picoline combination in *n*-butyronitrile as solvent (Scheme 7c) [61]. This electron-donating solvent and toluene in the former reaction acted as stabilizers to the electron-deficient silicon species in the similar mechanisms. First, the Brønsted or Lewis acid coordinates with silane **51** leading to a solvent-stabilized electron-deficient silane complex **57**, where *N*-protected indole attacks in a Friedel–Crafts fashion to give the 3-silylindoles **60** along with molecular hydrogen (Scheme 7b and Scheme 7d). A repetition of the processes leads to the bis(indol-3-yl)silanes **40**.

**Scheme 8:**
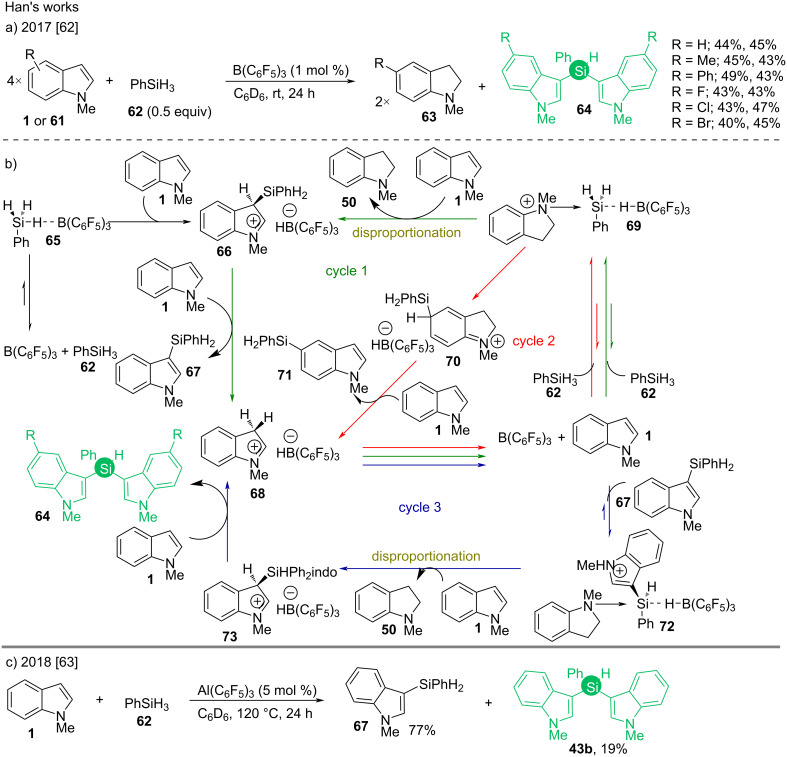
B(C_6_F_5_)_3_ and Al(C_6_F_5_)_3_-catalyzed syntheses of bis(indol-3-yl)silanes reported by Han.

Han described a Lewis acid-promoted C3-silylation of *N*-protected substituted indoles by a disproportionation mechanism of the latter. He used both B(C_6_F_5_)_3_ and Al(C_6_F_5_)_3_ in the reactions (Scheme 8a and Scheme 8c) which followed a similar mechanism (Scheme 8b) [62–63]. The reduced form of indole, i.e., indoline **50** coordinates with the Lewis acid to form a complex which activates PhSiH_3_ (frustrated Lewis pair) for silylation (**69**, Scheme 8b).

#### Phosphines

**Scheme 9:**
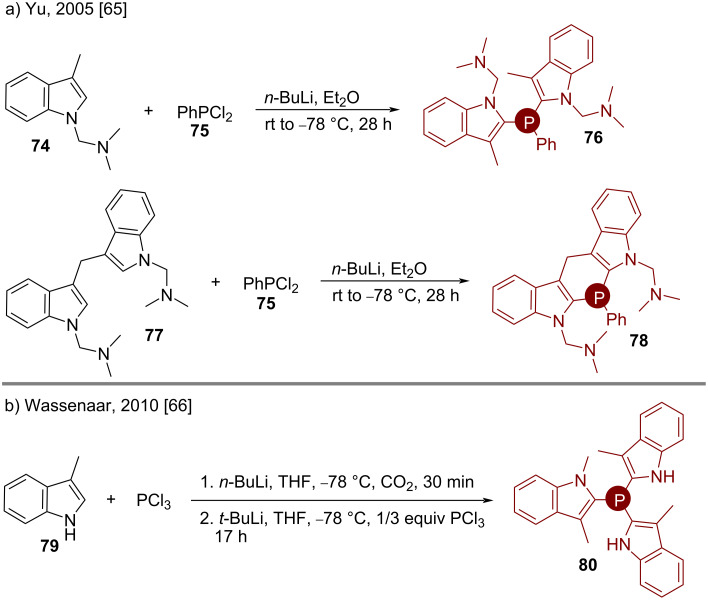
Base-mediated syntheses of bis and tris(indol-2-yl)phosphines.

The base-mediated syntheses of bis(indol-2-yl)phosphines **76** and **78** were demonstrated by Yu. A suitable halophosphine **75** was reacted with C2-deprotonated C3-tethered (**77**) or untethered (**74**) *N*-protected indoles for that purpose (Scheme 9a) [65]. Later, Wassenaar reported a similar strategy with trichlorophosphine as the electrophile for attaching three indole moieties to a single P-atom (**80**, Scheme 9b) [66]. A similar protocol was adopted by van de Watering in their recent syntheses [67–68].

#### Sulfides

The C2 tethering of indoles with sulfur can be achieved in neutral medium by treatment with various SL_2_ (L is a leaving group) moieties [69–70]. This is a common method for the synthesis of bis(indol-2-yl)sulfides which are the precursors of potent bioactive molecules [71–73].

**Scheme 10:**
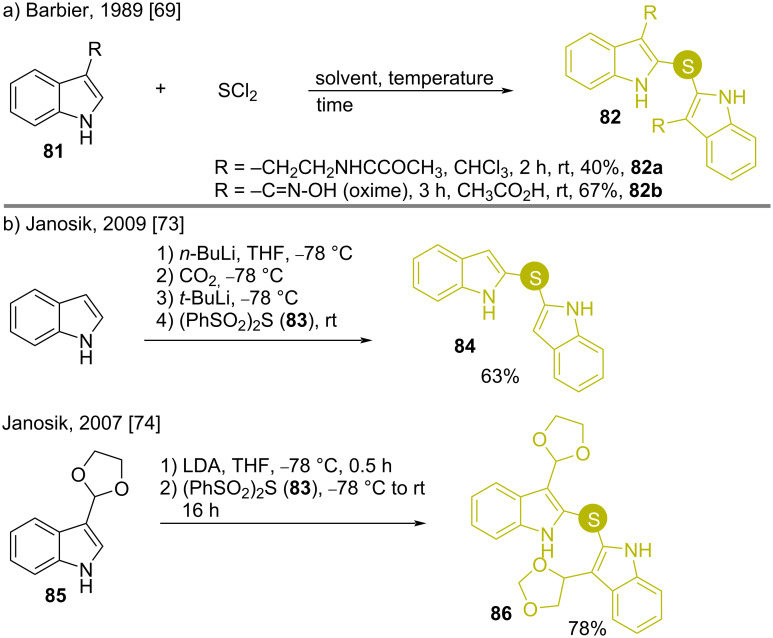
Synthesis of bis(indol-2-yl)sulfides using SL_2_-type reagents.

The simple synthetic strategies for the molecular units **82** were first reported by Barbier in 1989. The condensation of tryptamine monoacetate (**81a**) or indole oxime (**81b**) with sulfur dichloride in a Friedel–Crafts fashion (Scheme 10a) gave **82** with moderate to good product yields, respectively [69–70]. A similar work by Janosik involved strongly basic conditions at low temperature with bis(phenylsulfonyl)sulfide (**83**) as the sulfur donor (Scheme 10b) [73–74].

**Scheme 11:**
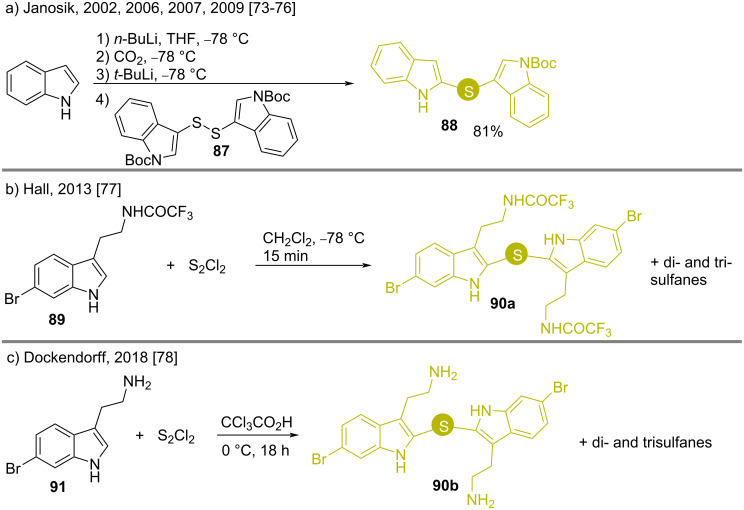
Synthesis of 2,3’- and 2,2’-bis(indolyl)sulfides using disulfides as substrates.

Disulfides are also important reagents for accessing bis(indolyl)sulfides. To synthesize the unsymmetrical bis(indolyl)sulfide **88**, Janosik reacted the indole disulfide **87** with free indole and obtained the product **88** in 81% yield, where the sulfur linkages were 2,3’- with respect to the two indole nuclei (Scheme 11a) [73–76]. Hall and Dockendorf prepared the corresponding 2,2’-sulfur-substituted compounds **90** by reacting tryptophan amines **89** and **90** with S_2_Cl_2_ under neutral and acidic conditions, respectively (Scheme 11b and Scheme 11c) [77–78].

**Scheme 12:**
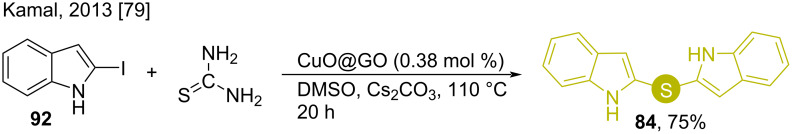
Synthesis of diindol-2-ylsulfide (**84**) from 2-iodoindole (**92**) and thiourea.

Kamal took a different approach using a CuO nanoparticle-supported graphene-oxide (denoted as CuO@GO, 0.38 mol %) catalyzed *S*-arylation (C–S coupling) of 2-iodoindole (**92**) to synthesize diindol-2-ylsulfide (**84**) in 75% yield (Scheme 12) [79]. Here 1.5 equivalents of thiourea acted as the sulfur source.

**Scheme 13:**
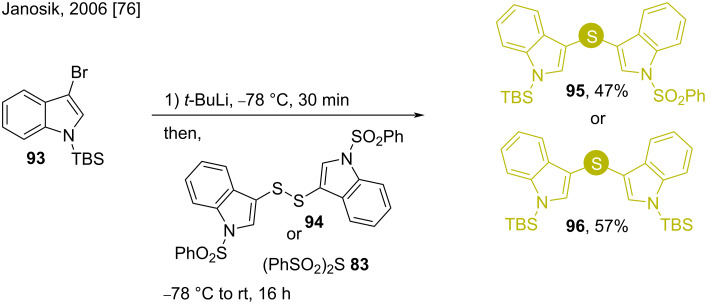
Synthesis of bis(indol-3-yl)sulfides using *N*-silylated 3-bromoindole **93**.

Bis(indol-3-yl)sulfides are also present as structural motifs in important organic compounds having semiconductor properties [80]. The syntheses of these compounds were studied by Janosik in 2006. The *N*-silyl-protected 3-bromoindole **93** was subjected to strong basic medium (*t*-BuLi) at low temperature and then quenched with either bis(phenylsulfonyl)sulfide (**83**) or indole disulfide **94** (Scheme 13) to afford the products **95** or **96**, respectively [76].

**Scheme 14:**
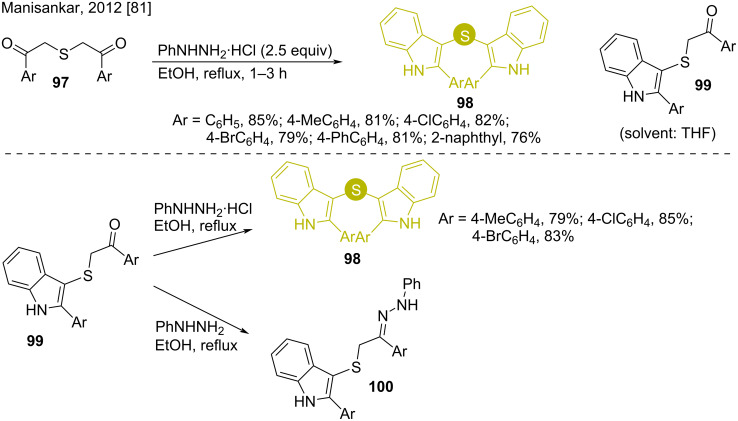
Fischer indole synthesis of bis(indol-3-yl)sulfides using thio diketones.

Manishankar and co-workers dealt with a facile Fischer indole process to convert thiodiketones **97** to bis(indol-3-yl)sulfides **98** by refluxing them with phenylhydrazine hydrochloride salt in ethanol [81]. Interestingly, changing the solvent to THF switched the product to thioketone **99** (Scheme 14). Refluxing the thioketones **99** again with phenylhydrazine hydrochloride in ethanol resulted in the desired bis(indol-3-yl)sulfides **98**. On the other hand, the treatment of thioketones **99** with phenylhydrazine afforded the corresponding hydrazones **100** only, thus stating the requirement of acid for this Fischer indole synthesis.

**Scheme 15:**
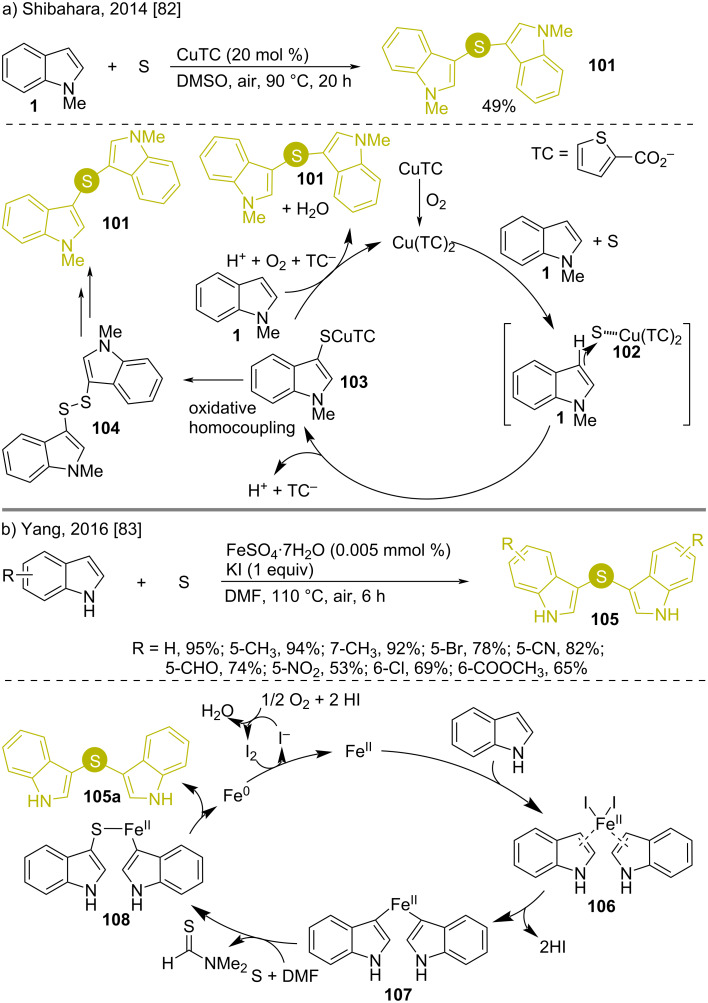
Oxidative synthesis of bis(indol-3-yl)sulfides using indoles and elemental sulfur.

Elemental sulfur has also been utilized in preparing bis(indol-3-yl)sulfides under transition-metal compound catalyzed spontaneous oxidation of the central chalcogen atom. Such reactions were carried out by Shibahara (2014) and Yang (2016) [82–83]. Both reactions used aerial oxygen as the oxidizing agent for sulfur (Scheme 15). Shibahara utilized 20 mol % copper(I) thiophene-2-carboxylate (CuTC) as the catalyst, where heating *N*-methylindole (**1**) with elemental sulfur in DMSO as solvent at 90 °C under aerial oxygen led to the desired product **101** in 49% yield [82]. Other copper catalysts such as CuCl or CuBr gave low yields, even when used with 2,2’-bipyridyl as the ligand. First, oxidation of copper(I) takes place, which interacts with elemental sulfur to “activate” it. A nucleophilic attack from *N*-methylindole (**1**) to the sulfur species **102** takes place to generate copper sulfide complex **103**. An oxidative homocoupling gives the bis(indol-3-yl)sulfide **101**. Simultaneously, an oxidative homocoupling of the copper sulfide complex can take place to afford disulfide **104**, that reacts with *N*-methylindole again under oxidative conditions, catalyzed by CuTC to give the desired product **101** (Scheme 15a).

On the other hand, Yang synthesized bis(indol-3-yl)sulfides **105** through the reaction of indole with elemental sulfur, catalyzed by iron(II) sulfate in the presence of stoichiometric amounts of KI in air [83]. The I^−^ from KI formed ferrous iodide, which reacts with indole to form the iron bis-indolide **107**, followed by reaction with *N*,*N*-dimethylmethanethioamide to get the S atom inserted (**108**). A reductive elimination then generated the bis(indol-3-yl)sulfides **105** along with Fe^0^, which was re-oxidized by aerial oxygen to re-participate in the reaction (Scheme 15b).

**Scheme 16:**
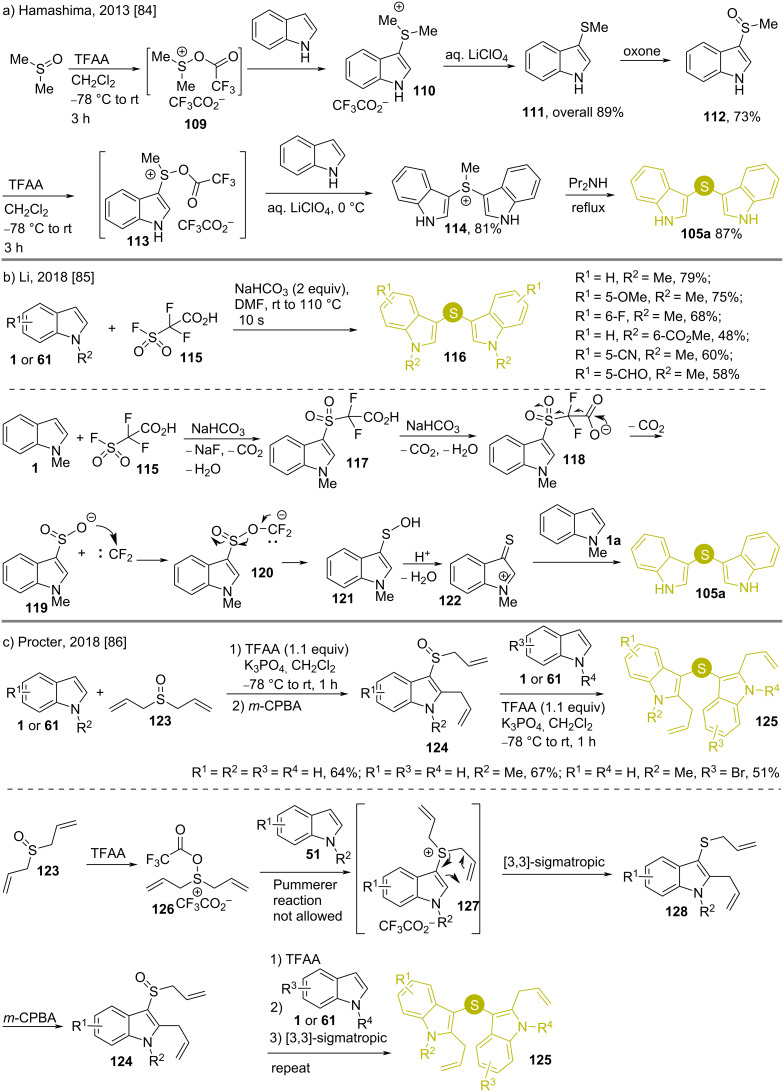
Synthesis of bis(indol-3-yl)sulfides using sulfoxides as sulfur source.

There are several uses of sulfoxides as a thiol-free sulfur source for introducing sulfur at the indole C3 position [84–86]. In 2013, Hamashima reported a synthesis of di(indol-3-yl)sulfide (**105a**) by reacting indole with DMSO in the presence of trifluoroacetic anhydride (TFAA) in total 6 steps (Scheme 16a) [84]. Already used by Hartke in 1988, this reagent combination (**109**) is a source for MeS^+^, so its use does not lead to any formation of disulfides [87]. First, **109** is attacked by indole and a demethylation of sulfur occurs leading to 3-(methylthio)indole (**111**). As the sulfur in **111** is methyl-protected, no dimerization occurs. Oxidation of sulfur by oxone followed by repetition of the previous steps afford the diindol-3-ylsulfonium salt **114**, which in the presence of a base gives product **105a**.

Li et al. used 2-(fluorosulfonyl)difluoroacetic acid (**115**) as the “S” source to synthesize bis(indol-3-yl)sulfides **116** from *N*-protected indoles **1** or **61** [85]. The products **116** were formed within a few seconds in the presence of a moderate base at high temperature (Scheme 16b), tolerating groups having both electron-donating and withdrawing nature on **1**. Here the base assisted the condensation of 2-(fluorosulfonyl)difluoroacetic acid (**115**) with **1** followed by decarboxylation to give difluorocarbene and sulfinate **119**, that combine to produce sulfanol **121**, which in the presence of acid and reaction with another molecule of indole affords **105**.

In 2018, Procter used a similar strategy to that reported by Hamashima for the synthesis of similar molecules **125** with good to moderate yields using electron-donating groups at the indole ring. The yields decreased with indoles having electron-withdrawing groups (Scheme 16c) [86]. Here diallyl sulfoxide (**123**) was used with TFAA to obtain diallyl intermediate **127**. The latter undergoes a [3,3]-sigmatropic reaction to afford allyl (2-allylindol-3-yl)sulfide **128**, which is oxidized by *m*-CPBA to sulfine **124**. Repetition of the steps along with indole addition led to the desired products. Here the absence of a β-hydrogen in the diallylsulfoxide (**123**) did not allow any Pummerer rearrangement [88–89].

#### Selenides

**Scheme 17:**
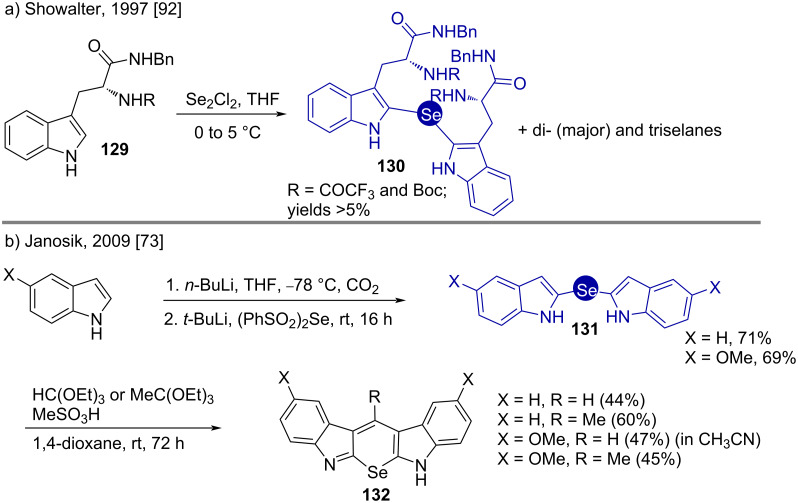
Syntheses of bis(indol-2-yl)selanes.

In 1997, Showalter synthesized bis(indol-2-yl)selanes (or selenides) **130** having potential tyrosine kinase inhibitor activities [90–91]. The synthesis was achieved by reacting diselenium dichloride with (*R*)-tryptophan amide **129** (Scheme 17a) [92]. Bis(indol-2-yl)selane **130** was found as a byproduct having very low such bioactivity. The polyselanes formed were separated by treating them with NaBH_4_, which did not affect the monoselane **130**.

On the other hand, selenopyrans structurally resemble indolocarbazoles, which possess AhR affinity [93]. Janosik presented a synthesis of such selenopyrans **132** via the bis(indol-2-yl)selanes **131** [73]. Treating these compounds with orthoformate esters in the presence of the Brønsted acid MeSO_3_H led to the target selenopyrans (Scheme 17b). The methylated analogs of **132** displayed high efficiency for activating AhR.

**Scheme 18:**
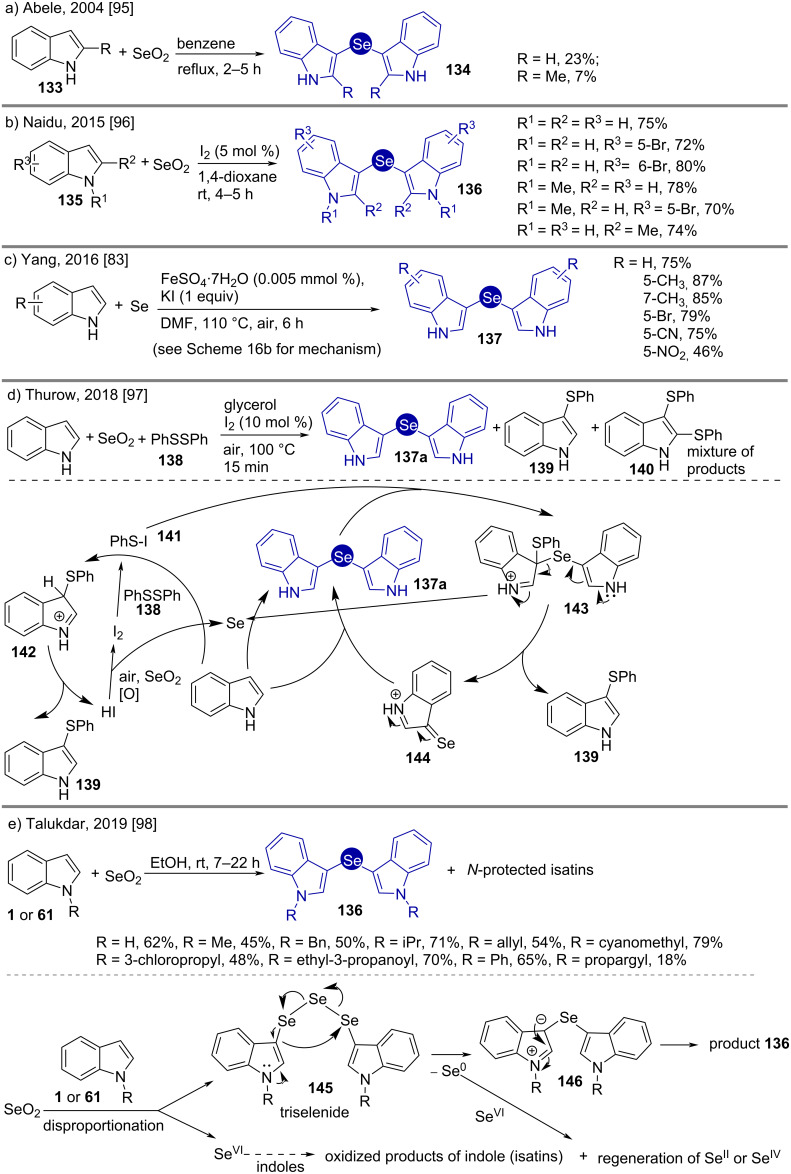
Syntheses of bis(indol-3-yl)selanes.

Bis(indol-3-yl)selanes possess antioxidant properties. Pioneered by Wilshire [94], their syntheses were studied by Abele [95], Naidu [96], Yang [83], Thurow [97], and Talukdar [98]. The work of Abele in 2004 involved refluxing SeO_2_ with *N*-unprotected indole in benzene which resulted in low yields of the products **134** (Scheme 18a) [44]. Using different *N*-protected substituted indoles **135**, Naidu observed improved yields of **136** when catalytic oxidant I_2_ was added in 1,4-dioxane as solvent (Scheme 18b) [96]. Using aerial oxygen as the oxidant, Yang used Se^0^ in the presence of stoichiometric KI and catalytic amounts of Fe^II^ for the synthesis of similar bis(indol-3-yl)selanes (Scheme 18c) [83].

In 2018 Thurow reported a method using stoichiometric SeO_2_ along with sub-stoichiometric PhSSPh (**138**) to obtain a mixture of the desired diindol-3-ylselane (**137a**) along with mono- and di(phenylthio)-substituted indoles **139** and **140** (Scheme 18d) [97]. Catalytic iodine was used to oxidize PhSSPh (**138**) to PhSI (**141**), to which indole adds to give (phenylthio)indole **139** along with HI. HI reduces SeO_2_ to Se. Se interacts with two molecules of indole in the presence of air to give the desired product **137a**. In a parallel pathway the product decomposes to selenone **144**, 3-(phenylthio)indole (**139**) and regenerates Se.

In a recent effort by Talukdar, the cheap and non-anhydrous solvent ethanol was used to prepare the desired bis(indol-3-yl)selanes **136** in moderate yields [98]. Following the assumption (formation of triselenide **145**) made by Wilshire [94] together with the detection of the oxidized products isatins in the reaction mixture, a disproportionation mechanism of SeO_2_ can be drawn giving bis(indol-3-yl)triselenide **145** and Se^VI^ (Scheme 18e). The triselenide **145** converts into bis(indol-3-yl)selane **146** with liberation of Se^0^. Se^VI^ can generate Se^II^ or Se^IV^ by either oxidizing indoles to isatins, or by a comproportionation reaction with Se^0^ to give **136**.

#### Tellurides

**Scheme 19:**
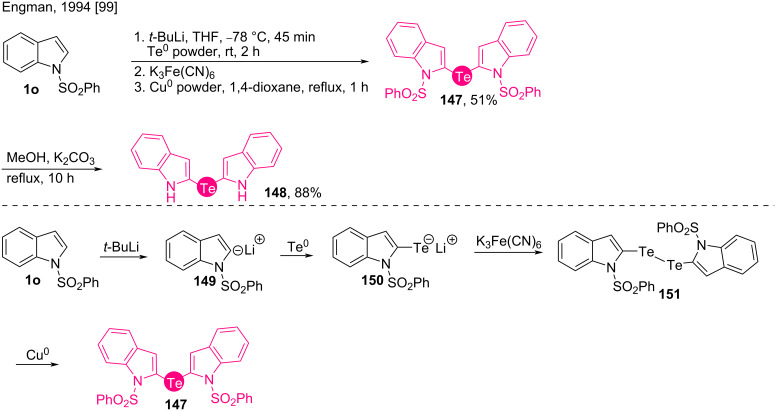
Synthesis of bis(indol-2-yl)tellane **147**.

Engman claimed a synthesis of the titular compounds **147** and **148** in the year 1994 by reacting the C2 anion **149** of the *N*-sulfonyl-protected indole **1o** with metallic Te in four steps including desulfonylation (Scheme 19) [99]. The treatment with base followed by the addition of elemental tellurium to N-protected indole **1o** generates lithium telluride **150**. Telluride **150** is then oxidized to ditelluride **151** by treatment with ferrocyanide. A Cu powder-mediated reduction gives the N-protected bis(indol-2-yl)tellane **147**. The final desulfonated product **148** is a potent thiol peroxidase reducing agent [100].

### The benzenoid C4 and C7 linkages

The syntheses of bisindolyl non-metallides connected through benzenoid rings of the indoles are less studied compared to the same through their pyrrole counterpart. The corresponding compounds are investigated for boron, nitrogen, oxygen, sulfur, and selenium as the central connecting atom.

#### Boranes

**Scheme 20:**
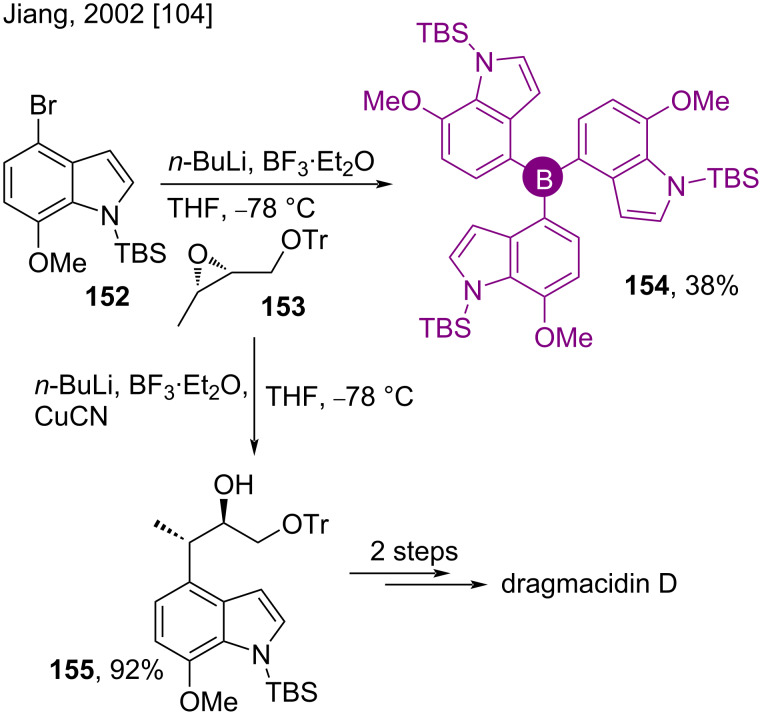
Synthesis of tris(indolyl)borane **154**.

The indole alkaloid dragmacidin D is a marine secondary metabolite which was recently found active against Parkinson’s and Alzheimer’s diseases [101–103]. In 2002, Jiang, while studying its synthesis, found the tris(indolyl)borane **154** instead of the desired chiral indole alcohol **155** while reacting the *N*-silylated 4-bromoindole **152** with *n-*BuLi in a failed regioselective ring opening attempt of chiral oxirane **153** in the presence of BF_3_**^.^**Et_2_O (Scheme 20) [104]. The synthetic route to the desired product was smoothly brought to its course by employing CuCN in the medium.

#### Amines

**Scheme 21:**
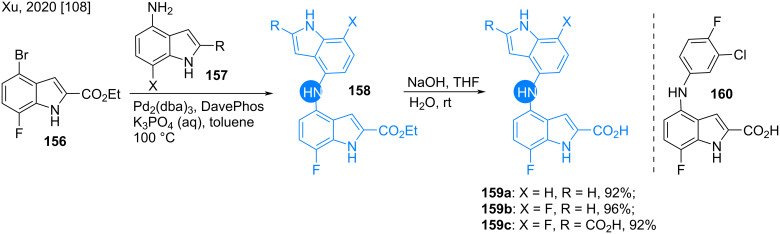
Synthesis of bis(indol-4-yl)amines **159**.

The enzymes indoleamine 2,3-dioxygenase 1 (IDO1) and tryptophan 2,3-dioxygenase (TDO) are responsible for tryptophan metabolism in the human body. Thus, the inhibition of these enzymes may help in tumor immunotherapy [105–107]. Xu recently found indole-2-carboxylic acid derivatives as IDO1/TDO dual inhibitors. In their effort to synthesize the following bis(indol-4-yl)amine derivatives via a Buchwald amination led to the 4-amino-substituted compounds **158** or acids **159** after basic hydrolysis (Scheme 21) [108]. Compound **159c** had the maximum potency against IDO1 and TDO with IC_50_ values of 2.72 mM and 3.48 mM, respectively compared to **159a** and **159b**, which is 15 and 28.5 times higher than that of hit compound **160**.

**Scheme 22:**
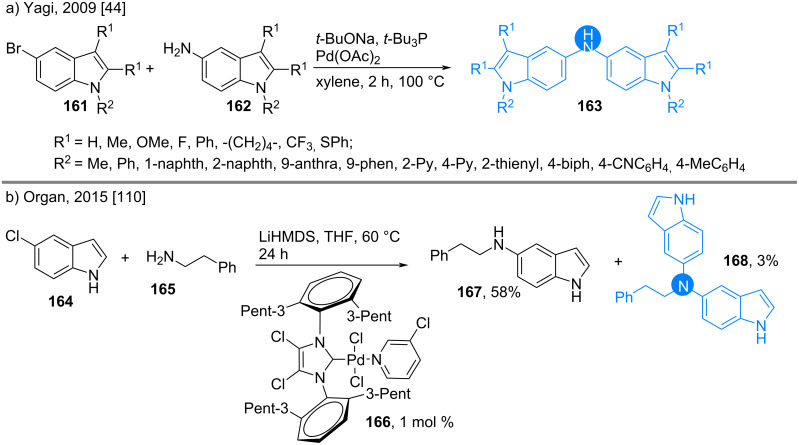
Synthesis of bis(indol-5-yl)amines.

As discussed earlier, bis(indolyl)amines possess electroluminescent properties [41,109]. In 2009, Yagi and co-workers synthesized a large library of bis(indol-5-yl)amines **163** for studying their efficiency in organic electroluminescent devices, where 5-bromoindoles and 5-aminoindoles were taken as partners in a Buchwald coupling (Scheme 22a) [44]. On the other hand, in 2015, Organ’s group performed a phosphine-ligand free Buchwald amination of 5-chloroindole (**164**) with amine **165** to give the desired product **167**, where the use of the Pd-PEPPSI-IPent^Cl^ precatalyst **166** in presence of the strong base led to the formation of the over-aminated product **168** (Scheme 22b) [110].

**Scheme 23:**
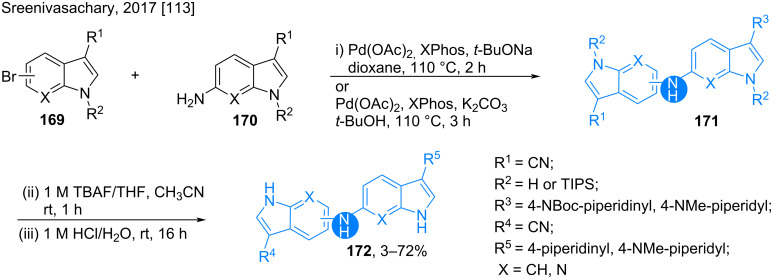
Synthesis of 6,5’/6,6’-bis(indolyl)amines.

Alzheimer’s disease is caused by the β-amyloid-42 aggregation in brain tissue [111–112]. In 2017, Sreenivasachary synthesized a library of 6,5’- and 6,6’-bis(indolyl)amines and other similar 7-azaindole derivatives as potent anti-Alzheimer agents (**171**, **172**) by a Buchwald coupling of the corresponding C3-substituted amines **170** and indole 5/6-bromides **169** (Scheme 23) [113]. Cyano, 4-piperidinyl and *N*-methylpiperidinyl substitutions at the indole and 7-azaindoles were necessary to improve the brain penetration ability of the products.

Up to >80% inhibition of the amyloid-β peptide aggregates were achieved with these compounds, with the highest activity found for the 4-*N-*methylpiperidyl derivative.

#### Ethers

**Scheme 24:**
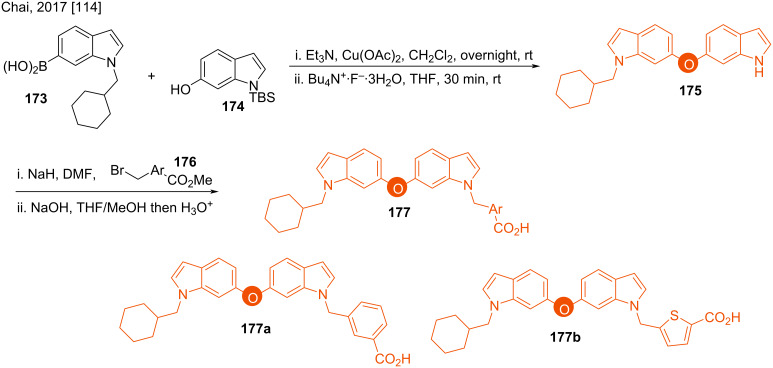
Synthesis of potent HIV-inhibitors 6,6’-bis(indolyl) ethers.

The synthesis of the bis(indol-6-yl) ether **175** was performed by Chai in 2017. Their protocol used a Cu(OAc)_2_-mediated coupling of *N*-silylated 6-hydroxyindole **174** with the corresponding boronic acid **173** (Scheme 24) [114]. For further synthetic transformations of **175**, *N*-protection with bromo esters **176** followed by hydrolysis towards acids **177a** and **177b** were performed. The products **177a** and **177b** are potent anti-HIV agents.

**Scheme 25:**
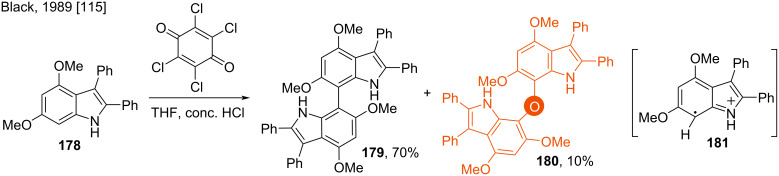
Synthesis of bis(indol-7-yl) ether.

Although the synthesis of 7,7’-bis-indolyl ether was known prior to Chai’s report [114]. In 1989, Black found the 7,7’-dimerised product **179** of the indole derivative **178** as a hindered biphenyl analog via its prompt oxidation in the presence of quinones. The bis(indol-7-yl) ether **180** was found in 10% yield when chloranil was used as the oxidant (Scheme 25) [115]. The high electrophilicity of **178** at the C7 position resulted in this product formation. The reaction proceeds through the radical intermediate **181**.

#### Sulfides

**Scheme 26:**
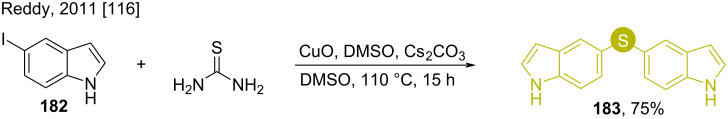
Synthesis of di(indol-5-yl)sulfide (**183**).

Reddy synthesized the di(indol-5-yl)sulfide (**183**) via a cascade strategy with 5-iodoindole (**182**) in the presence of thiourea and a recyclable CuO nanoparticle catalyst (Scheme 26) [116]. This heterogeneous catalysis strategy bypasses the use of unpleasant aryl thiols, which are generally coupled with other aryl halides in the presence of transition-metal catalysts for obtaining diaryl sulfides [117].

#### Selenides

**Scheme 27:**
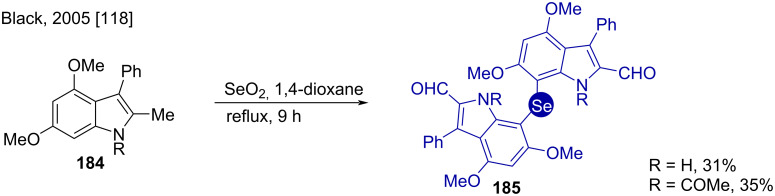
Syntheses of 2,2’-diformyl-7,7’-bis(indolyl)selenides.

Along with the oxygen insertion, Black et al. also performed the oxidative selenium insertion into the C-7 position of highly electrophilic 2-methylindole derivative **184**. The dual role of selenium dioxide consists of activation of the C-7 position giving the dimerized 7,7’-bis(indolyl) products **185** with the 2-methyl group transformed to the aldehyde in the same step (Scheme 27) [118–119]. The less electronically activated *N*-acyl substrate gave a slightly better yield. Selenation occurs at C-3 instead of C-7 for the C-3 unsubstituted substrates.

## Conclusion

This review summarizes the various (un)catalytic synthetic techniques of the symmetric and unsymmetric bis/tris(indolyl)-containing non-metallides consisting of multiple indole molecules covalently connected via C2, C3 (pyrrole ring) and C4–C7 (benzenoid ring) by different central atoms. Like the bis(indolyl)methanes (anticancer substances), these products are important potential pharmaceutically active ingredients as well. As a result, they have gathered much attention in the current decade as suggested by the number of contemporary publications associated. The described schemes involve both simple and challenging strategies depending on the central tethering atom involved. As time progresses, research on the synthesis and application of this class of molecules will be more broadened.
